# Pharmacological Approach for Neuroprotection After Cardiac Arrest—A Narrative Review of Current Therapies and Future Neuroprotective Cocktail

**DOI:** 10.3389/fmed.2021.636651

**Published:** 2021-05-18

**Authors:** Rishabh C. Choudhary, Muhammad Shoaib, Samantha Sohnen, Daniel M. Rolston, Daniel Jafari, Santiago J. Miyara, Kei Hayashida, Ernesto P. Molmenti, Junhwan Kim, Lance B. Becker

**Affiliations:** ^1^Laboratory for Critical Care Physiology, The Feinstein Institutes for Medical Research, Northwell Health, Manhasset, NY, United States; ^2^Department of Emergency Medicine, Northshore University Hospital, Northwell Health, Manhasset, NY, United States; ^3^Donald and Barbara Zucker School of Medicine at Hofstra/Northwell, Hempstead, NY, United States; ^4^Department of Anesthesiology, Dartmouth-Hitchcock Medical Center, Lebanon, NH, United States; ^5^Department of Surgery, North Shore University Hospital, Northwell Health, Manhasset, NY, United States; ^6^Elmezzi Graduate School of Molecular Medicine, Manhasset, NY, United States; ^7^Department of Surgery, Northwell Health, Manhasset, NY, United States

**Keywords:** cardiopulmonary arrest, ischemia and reperfusion injury, resuscitation, neuroprotection, cerebral ischemia, pharmacological intervention, cocktail therapy

## Abstract

Cardiac arrest (CA) results in global ischemia-reperfusion injury damaging tissues in the whole body. The landscape of therapeutic interventions in resuscitation medicine has evolved from focusing solely on achieving return of circulation to now exploring options to mitigate brain injury and preserve brain function after CA. CA pathology includes mitochondrial damage and endoplasmic reticulum stress response, increased generation of reactive oxygen species, neuroinflammation, and neuronal excitotoxic death. Current non-pharmacologic therapies, such as therapeutic hypothermia and extracorporeal cardiopulmonary resuscitation, have shown benefits in protecting against ischemic brain injury and improving neurological outcomes post-CA, yet their application is difficult to institute ubiquitously. The current preclinical pharmacopeia to address CA and the resulting brain injury utilizes drugs that often target singular pathways and have been difficult to translate from the bench to the clinic. Furthermore, the limited combination therapies that have been attempted have shown mixed effects in conferring neuroprotection and improving survival post-CA. The global scale of CA damage and its resultant brain injury necessitates the future of CA interventions to simultaneously target multiple pathways and alleviate the hemodynamic, mitochondrial, metabolic, oxidative, and inflammatory processes in the brain. This narrative review seeks to highlight the current field of post-CA neuroprotective pharmaceutical therapies, both singular and combination, and discuss the use of an extensive multi-drug cocktail therapy as a novel approach to treat CA-mediated dysregulation of multiple pathways, enhancing survival, and neuroprotection.

## Introduction

Annually, there are over 356,000 out-of-hospital cardiac arrests (OHCA) and 209,000 in-hospital cardiac arrests (IHCA) in the United States with the survival to hospital discharge only 12% and 25%, respectively. Furthermore, only 8.4% of OHCA patients have favorable neurologic function, which can be determined using various scales, such as modified Rankin Scale (mRS), Cerebral Performance Categories (CPC), and Extended Glasgow Outcome Scale (1–3). Despite the heterogeneous etiologies of cardiac arrest (CA), the commonality is the decreased perfusion to vital organs, such as the brain (4). Hence, the best chances for increasing survival and clinical outcomes requires a combination of early recognition, early initiation of high-quality cardiopulmonary resuscitation (CPR) and Advanced Cardiovascular Life Support (ACLS), and appropriate post-resuscitation care that focus on protecting the brain (5). Despite proven benefits of these interventions on survival, the vast majority of survivors still have significant neurologic deficits; therefore, novel neuroprotective therapies that may be achieved through combination of evidence-based, pathway-specific, pharmacological agents can potentially improve outcomes for patients.

Ischemia-reperfusion injury (IRI) begins with decreased perfusion to tissues, depletion of energetic substrates, and subsequent upregulation of the anaerobic metabolism (6). Under physiological conditions, the brain consumes 20% of the basal metabolic rate despite representing only 2% of the total body weight. This high energetic demand, the virtual absence of glycogen reserves, and the dependance on oxidative phosphorylation makes the brain highly vulnerable (7, 8). Resuscitation with return of spontaneous circulation (ROSC) increases the chance of overall survival, however the subsequent reperfusion injury has been associated with further neurological damage (3).

Besides ACLS, there are limited evidence-based treatments that successfully improve survival and protect the brain after CA. The two major therapies used for treating CA that can aid in neuroprotection are extracorporeal cardiopulmonary resuscitation (E-CPR) and targeted temperature management (TTM); the former uses an external machine to oxygenate and circulate blood within the body (9), while the latter decreases and maintains the core temperature between 32 and 36°C to decrease cellular metabolism, decrease oxidative damage and stress signals, and decrease cellular death (6, 10–13). Although both E-CPR and TTM have therapeutic efficacy in CA patients, each has their limitations and can influence both protective and deleterious pathways (14). E-CPR is time, personnel, and resource intensive. Major complications of TTM include hemodynamic instability, arrhythmias, AV blocks, hydro-electrolyte disorders due to cold diuresis, endocrine and coagulation abnormalities, increased risk of infection, among others (15, 16).

Although improvements in technology may increase the general application of TTM and E-CPR, the major challenge resides in tackling the various molecular mechanisms that result in brain injury post-CA (14). Currently, preclinical and a few clinical studies have used individual pharmacologic agents to alleviate damage post-CA, yet these interventions are unable to provide sufficient protection to aid in survival with favorable neurological outcomes. As such, the focus of this review is to highlight therapies that have attempted to confer neuroprotection post-CA in preclinical and clinical studies, with their proposed targeted pathway(s). We also seek to propose the application of a multi-drug cocktail that is comprised of extensive pharmaceutical agents rather than a few in order to concurrently target the distinct, multiple metabolic, oxidative, and inflammatory alterations in the brain post-CA to potentially shift the current paradigm of neuroprotection to a multidimensional therapeutic approach.

## Overview of the Various Cardiac Arrest-Mediated Pathway Alterations

**Figure 1:**
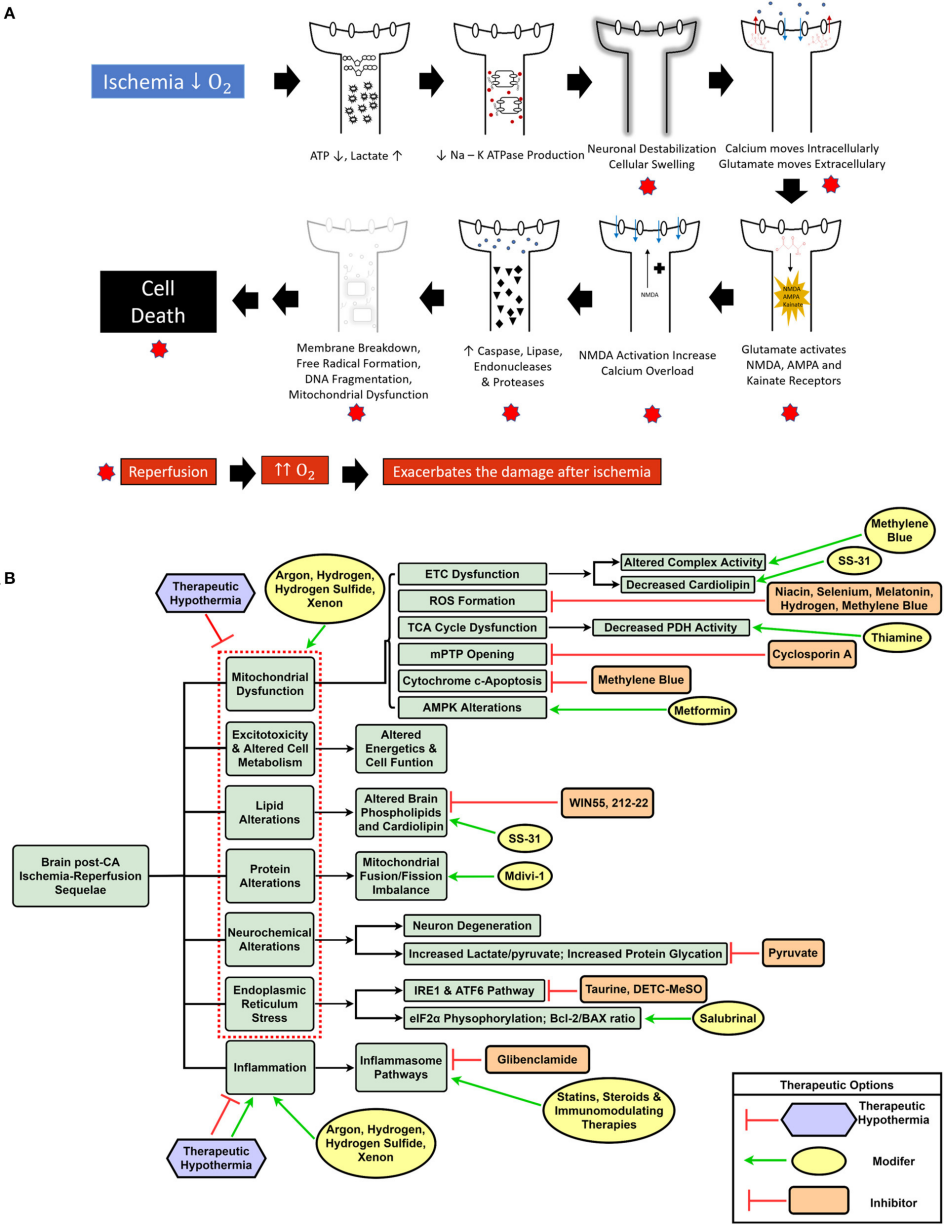
Schematic representation of ischemia-reperfusion injury after cardiac arrest and resuscitation resulting in brain injury **(A)**. Overview of dysregulated pathways after cardiac arrest with a selection of inhibitory and modulatory interventions that can be combined in a cocktail therapy to confer neuroprotection **(B)**.

CA and the ensuing global ischemia generate a variety of alterations, such as mitochondrial dysfunction, increased reactive oxygen species (ROS) generation (17, 18), metabolic disruption including protein and lipid dysfunction (19–23), increased neuroinflammation, intracellular calcium overload, and endoplasmic reticulum (ER) stress (24–26). Ultimately, CA pathologic alterations result in cellular dysfunction and brain injury of which substantial mitigation necessitates a change in the current paradigm of CA treatment from targeting singular altered pathways to using a multifunctional, multi-drug cocktail method (14). Figure 1A summarizes the various mechanisms of brain damage post-CA.

### Cardiac Arrest-Mediated Mitochondrial Dysfunction and ROS Generation With the Respective Therapeutic Interventions

Mitochondrial dysfunction has been correlated with poor neurologic outcomes secondary to impaired aerobic metabolism as well as increased oxidative stress, garnering attention as a target for neuroprotection after CA (17, 27, 28). Along with the general inefficiency in mitochondrial energy generation (19), CA patients exhibit low activity of pyruvate dehydrogenase (PDH) complex, the rate-limiting step in the Tricarboxylic acid (TCA) cycle (27, 29). Therefore, administering thiamine, a cofactor of the PDH complex, to mice after CA resulted in improved PDH activity and neurological outcomes (27). Decreased ATP production and impairment of the Na^+^/K^+^ ATPase (6) after CA results in intracellular calcium overload triggering the voltage-dependent anion channels (VDAC) and subsequent opening of the mitochondrial permeability transition pore (mPTP) (30, 31). Formation of the mPTP further activates the caspase cascade and endonuclease-mediated DNA damage resulting in mitophagy and cellular death (30). Cyclosporin A has been studied to provide neuroprotection by preventing mPTP opening both alone and in combination with hypothermia in rats, but human trials have not supported the therapeutic benefits observed in the preclinical setting (32–34).

The disruption of mitochondrial fusion and fission equilibrium has been documented in rats after CA, with temporary mitochondrial fusion shortly following CA, which shifts toward fission within 24 h, resulting in deficient cellular and mitochondrial function and eventual apoptosis (35). Inhibiting mitochondrial fission has been shown to prevent cardiomyocyte apoptosis and improve cardiac function *in vitro, ex vivo*, and *in vivo* (36, 37). Inhibition of the mitochondrial fission protein, Dynamin-related protein 1 (Drp1), using the well-known fission inhibitor, mitochondrial division inhibitor 1 (Mdivi-1), has shown to improve neurological dysfunction and survival in mice after CA (38, 39), prevent N-Methyl-d-aspartate (NMDA)-mediated excitotoxicity, calcium overload, cell death in rat neurons (40), and has cardioprotective properties in a rat model of cardiac IRI (41). Taken together, CA results in major metabolic alterations, including mitochondrial protein damage, that require targeting multiple pathways simultaneously for improved survival and neuroprotection.

Post-CA mitochondrial dysfunction generates ROS due to enhanced electron leak at complexes I and III in the electron transport chain, deglutathionylated complex II, and phosphorylated complex IV, among other mechanisms (42). ROS-mediated release of cytochrome c from the mitochondrial membrane upregulates pro-apoptotic genes causing apoptosis (30). General application of antioxidant therapies, such as vitamin C, α-tocopherol, and edaravone, have shown attenuation of ROS in preclinical and some clinical settings (43–45). Although there are multiple ongoing clinical trials evaluating the beneficial effects of antioxidant therapies in human CA patients, the mixed beneficial evidence from the preclinical setting does not support the translation of these singular antioxidant therapies (46–48). Site-specific inhibitors of ROS are more promising candidates because (1) they directly control the release of ROS at the source and (2) ROS generation at different sites can play either a beneficial, or a detrimental role (49). N-acetylcysteine, metformin, melatonin, and suppressor of site IQ electron leak (S1QEL) are a few examples of site-specific inhibitors that can decrease ROS generation after IRI and can help to potentiate survival and neurological functions (50–53). Inhaled hydrogen gas with its suppression of oxidative stress has also been observed to improve survival and neurologic outcomes in a rat CA model with an additive effect when compared to TTM alone (54). Similarly, other inert gases, such as Argon and Xenon seem to potentiate beneficial effects from standard treatments post-CA *via* a variety of proposed mechanisms (55–59). Mitochondrial dysfunction after IRI results from alterations in multiple pathways and further induces substantial metabolic and cellular disruption; therefore, utilizing a multi-drug cocktail that incorporates these therapies can target numerous mitochondrial pathways and may improve survival and brain function.

### Cardiac Arrest-Mediated Endoplasmic Reticulum Stress With the Respective Therapeutic Interventions

Endoplasmic reticulum stress after intracellular calcium disruption or accumulation of unfolded/misfolded proteins in the ER lumen leading to the unfolded protein response (UPR) can be secondary to oxygen and glucose deprivation (30, 60). This, in turn, can expedite neurodegeneration through suppression of protein synthesis, protein degradation, and apoptosis (60). In a rat model of cerebral ischemia, the combination of taurine and S-Methyl-N, N-diethylthiocarbamate sulfoxide (DETC-MeSO), a partial NMDA antagonist, resulted in decreased expression of multiple ER stress pathways, which was insufficient when only using the individual therapies (61). Pretreatment using Salubrinal 30 min before CA improved neurological outcomes and cerebral mitochondrial morphology 24 h after CA by preservation of the mitochondrial membrane potential, increasing an antiapoptotic protein, Bcl-2, stabilizing the HIF-1α pathway, and inhibiting ER stress through the induction of eIF2α phosphorylation (62). Overall, these studies suggest that interventions targeting ER stress are important for neuroprotection post-CA and should be part of a larger cocktail therapy.

### Cardiac Arrest-Mediated Metabolic Alterations and Therapeutic Interventions

Many studies have demonstrated the substantial and global metabolic changes that occur after CA and resuscitation. In a model of long duration CA and cardiopulmonary bypass (CPB) resuscitation, the severe metabolome dysregulation in the kidney and brain worsened post-resuscitation, as seen through alterations of fatty acids, amino acids, and TCA cycle metabolites (19). Kynurenine pathway alterations have been observed in rat, pig, and human plasma after CA, while in rats and pigs, higher levels of downstream metabolites were associated with worse survival and neurological outcomes observed through hippocampal lesions (20, 63). A study on plasma metabolomic profiling in pigs undergoing asphyxial cardiac arrest (ACA) or ventricular fibrillation cardiac arrest (VFCA) further supported major alterations in the plasma metabolites related with TCA and urea cycles (64). In a rat model of CA, pre-conditioning with metformin demonstrated early and sustained 5′-adenosine monophosphate-activated protein kinase (AMPK) activation in hippocampal brain tissue, reduced neuronal death, and improvement in overall survival with favorable neurologic outcomes (65). However, the role of AMPK after IRI has been controversial as a focal brain ischemia model suggested that downregulating AMPK may show better outcomes (66). AMPK is a mediator of metabolism and is involved in many cellular mechanisms (67) suggesting its role after different types of IRI pathologies and varying degrees of injury may either facilitate neuroprotection or participate in injury. This is an active area of exploration.

Along with individual metabolic alterations post-CA, specific changes in lipids and proteins are observed that implicate CA-induced mitochondrial injury. Lipids comprise a substantial category of biological materials that have unique and crucial functions in the body, such as cellular membrane composition, energy substrates and metabolites, transport mediators, as well as intracellular and hormonal signaling (68, 69). Free radical-mediated lipid peroxidation and decomposition of membrane phospholipids occurs after IRI (70, 71). Dogs undergoing 10 min CA had significantly increased levels of lipid oxidation in the frontal cortex, which continued to increase until 24 h post-resuscitation when ventilated with 100% oxygen; normoxic ventilation significantly lowered lipid oxidation and improved neurologic outcomes (21, 72). CA induces phospholipid changes in the brain, heart, kidney, and liver of rats post-CA with the brain having increased concentrations of lysophosphatidylethanolamine (LPE), lysophosphatidylcholine (LPC), and lysophosphatidylinositol (LPI), whereas only LPI was increased in the other organs; the brain's inability to regulate phospholipids could be related to decreased mitochondrial function (73–75). Decreased levels of brain cardiolipin (CL) post-CPB resuscitation in rats adversely affected mitochondrial integrity and decreased normal function resulting in decreased complex I and III activity in the brain (76). Plasma accumulation of brain-specific cardiolipin species after CA may represent the degree of neuronal injury after human and rat CA (77), while changes in mitochondrial lipids, phospholipids, and free fatty acids were noted after lethal ventricular tachyarrhythmia-mediated ischemia in rats (78). Administration of SS-31, a CL-targeting drug, in a severe CA rat model resulted in lower lactate levels and improved survival time, suggesting that improved support of CL can improve mitochondrial function (79). Furthermore, treatment with a cannabinoid receptor agonist, WIN55, 212-2, in combination with TTM was able to support lipid metabolism and improve neurologic outcomes after rodent CA (80). The use of probucol, a lipid-lowering agent used for hypercholesterolemia (81), increased survival time and decreased oxidative stress in post-CA rats potentially through antioxidant mechanisms (82). Collectively, these studies suggest that utilization of drugs that are lipid-based and/or target lipid pathways are vital components of a multi-drug cocktail for the treatment of CA.

### Cardiac Arrest-Induced Neurochemical and Neuroinflammatory Alterations With Their Respective Therapeutic Interventions

Following cerebral ischemia, the acute efflux of dopamine and norepinephrine propagate brain injury (83), with the potential to significantly damage cerebral nerve terminals (84). Additionally, dopamine is considered a prerequisite for ischemic injury, since dopamine depletion protects the striatum from ischemic injury in a rat model of global brain ischemia by four-vessel occlusion (85). It is postulated that metabolic degradation of dopamine produces 2,4-dihydroxyphenylacetic acid (DOPAC), hydrogen peroxide, superoxide, and hydroxyl radicals, which have varying degrees of neurotoxic effects (86). In a small cohort of CA human patients undergoing TTM, lactate/pyruvate ratio measured using cerebral microdialysis progressively increased in the group of patients with an unfavorable neurologic outcome (87). In a swine model of CA, administration of intravenous pyruvate that can function as an antioxidant helped to minimize CA-induced protein glycation in the brain (88). Thus, the resultant neurochemical changes after IRI provide specific targets for therapeutic interventions.

Cerebral ischemia results in increased release of proinflammatory cytokines, such as IL-1 and TNF-α, microglial activation, disruption of the blood-brain barrier (BBB) and subsequent cerebral edema, all facilitating leukocyte migration into the brain (6, 83). Directly targeting inflammatory cytokine receptors is neuroprotective, as seen with reduced cortical infarct size after mild hypoxia/ischemia in knockout mice for IL-1-receptor (89) and with diminished brain injury in rats after blocking TNF-α *via* a neutralizing antibody (90). A majority of the neuroinflammation post-ischemia is microglia- and peripheral leukocyte-mediated (91). IRI stimulates IL-1β, IL-18, and NF-κB, activating the NLRP3 inflammasome, caspase-1, and other downstream mediators resulting in cell death and brain damage (92). Glibenclamide (GBC), a sulfonylurea drug used for diabetes, has been shown to inhibit the NLRP3 inflammasome pathway, suppress microglia and astrocyte activation, and support favorable neurologic outcomes following rodent CA (93). Furthermore, the immunomodulatory and antioxidative properties of statins have also shown efficacy in ischemic diseases (94). Thus, targeting the various pathways involved in neuroinflammation post-CA is a valuable therapeutic option, especially when used in a cocktail therapy.

## Current Combination Therapies Applied for Conferring Neuroprotection After Cardiac Arrest

A detailed compilation of various pharmaceutical interventions attempted after CA in the preclinical and clinical settings along with their proposed mechanisms of actions and potential disadvantages is discussed in Table 1. Previous studies have examined some neuroprotective drugs in cerebral ischemia and CA (57, 58, 83, 107). While these studies have occasionally examined drug combinations, the drugs mentioned often target a singular pathway among the background of a global disease, potentially explaining the lower effectiveness of these therapies.

**Table 1:** Pharmacological interventions with their study results, mechanisms of action, and potential drawbacks for conferring survival and neuroprotection after cardiac arrest.

**References**	**Drug(s) studied**	**Study model and delivery route**	**Pathway impacted**	**Study results**	**Drawbacks**
Katz et al. (95)	HBN-1 (ethanol, epinephrine, and vasopressin)	Rat/IV	Pharmacologically induced hypothermia	Decreased time to reach target temperature, improved survival, improved NDS	Mechanism of action not clear
Argaud et al. (33) Knapp et al. (34) Liu et al. (32)	Cyclosporine Cyclosporine A (CsA) Cyclosporine A (CsA) and Hypothermia	Human/IV Rat/IV Rat/IV	mPTP pathway mPTP pathway mPTP pathway	OHCA patients with non-shockable rhythms did not show improvement in outcomes or neurological status Non-statistically significant improvement in neurological tests and outcomes Mitochondrial membrane stabilization, apoptosis inhibition, ROS mitigation; synergistic effects of CsA and hypothermia	Unclear interaction with TTM; ideal timing of drug administration still unknown Animals kept normothermic; no examination of nephrotoxicity of immunosuppression End point only 2 h after resuscitation; did not study side effects of CsA; only studied one CsA dose
Cariou et al. (96) Cariou et al. (97)	Erythropoietin Erythropoietin and Hypothermia	Human/IV Human/IV	Erythropoietin-mediated pathways and mechanism not discussed Erythropoietin-mediated pathways and mechanism not discussed	OHCA patients resuscitated from presumed cardiac cause, early administration of erythropoietin plus standard therapy did not confer a benefit, and was associated with a higher complication rate OHCA patients resuscitated with administration of erythropoietin plus hypothermia demonstrated non-statistically significant increased survival rate	Significant adverse effects were observed with no benefits to survival with minor neurological sequelae Hematologic adverse events were observed, non-statistically significant increased survival rate, and no significant difference observed in neurological recovery
Ikeda et al. (98)	Estrogen	Mice/IV	Mechanism not discussed	Increased kidney protection in male and aged female mice	Did not show survival difference, neurological outcomes, and lacked mechanism
Huang et al. (99) Yang et al. (93)	Glibenclamide (GBC) Glibenclamide (GBC)	Rat/IP Rat/IP	SUR1-TRPM4 channel NLRP3 inflammasome pathway	GBC comparable to TTM in improving both survival and neurologic outcomes, suppressed activation of microglia and astrocytes, hypoglycemia not detected GBC improved electrophysiological recovery and neurological functional outcome	Mechanism of action incompletely understood—did not prove causal relationship with SUR1-TRPM4 channel Mechanism of action incompletely understood
Scott et al. (88)	Pyruvate	Pig/IV	Attenuating mitochondrial dysfunction	Preserved multiple enzyme systems that protect the brain from glycation stress	Specific glycated proteins not yet identified; endpoint was only 4 h after cardioversion and ROSC; pyruvate may have limited use clinically due to side effect of hypocalcemia
Li et al. (100)	Methylene blue and therapeutic hypothermia	Rat/SQ	Therapeutic hypothermia and attenuating mitochondrial dysfunction	Combination yielded markedly higher number of surviving neurons and reduced cognitive deficits	Higher doses has significant side effects, such as cardiovascular effects, headaches, vomiting, diarrhea, blue urine, epidermal damage, serotonin syndrome in those taking selective serotonin reuptake inhibitors, and anemia in those with glucose-6-phosphate dehydrogenase deficiency
Yang et al. (52)	Melatonin	Rat/gavage	ROS production	Pre- and post-treatment can help improve neurologic deficits and improve cognitive function after CA/CPR	Oral gavage had high dose due to relatively low bioavailability as compared to intraperitoneal or intravenous injection; mechanism of action not completely understood
Zhu et al. (65)	Metformin	Rat/intragastrically	AMPK-induced autophagy	Pre-treatment resulted in increased 7-day survival with significantly improved NDS; post-arrest treatment ameliorated histological injury and neuroinflammation	AMPK pathway incompletely understood post-CA–activation effects may depend on stimulus and duration
Wiklund et al. (101) Miclescu et al. (102)	Methylene Blue and postponed hypothermia Methylene Blue	Pig/IV Pig/IV	Antioxidant, nitric oxide inhibitor, and participates in electron shuttling in mitochondria Decreasing nitric oxide metabolism	Reduced cerebral cortical neuronal injury and blood–brain barrier disruption after methylene blue with postponed hypothermia Protected blood brain barrier	Did not measure survival and mechanism is not completely understood Did not measure survival or neurological function; mechanism is not completely understood
Zhang et al. (79)	SS-31	Rat/IV	Mitochondrial inner membrane stabilization- cardiolipin	Lowered lactate levels and improved survival rate 5 h after 25 min CA and 30 min CPB resuscitation	Mechanism of action incompletely understood
Zhang et al. (62)	Salubrinal	Rat/IP	ER stress and mitochondrial stabilization	Improved neurological performance and mitochondrial morphology 24 h after CA and resuscitation	Treatment was prior to CA induction and resuscitation; only one dose tested; only one end point of 24 h
Bar-Joseph et al. (103)	Sodium bicarbonate	Human/IV	Mechanism not discussed	Administration was associated with higher early resuscitation rates with better long-term outcome	Dose-dependence was observed: low dose (1 mEq/Kg) was beneficial as compared with high dose (>1 mEq/Kg)
Ikeda et al. (27)	Thiamine	Mice/IV and IP	PDH modulation in the TCA	Improved neurologic outcome and 10-day survival	Impact on other organs not examined
Tsai et al. (104) Katz et al. (105)	Corticosteroid Corticosteroid	Human/IV Rat/IV	Altering the inflammatory cascade and microcirculatory flow Decreased brain enzyme changes and decreased requirement for vasopressor	Improved survival to discharge in human patients Enhancing cardiovascular and EEG recovery	Did not influenced brain enzyme levels at 20 min post-CA
**Gases**					
Tamura et al. (106)	Hydrogen	Human/inhalation	Mechanism not discussed	Efficacy of inhaled HYdrogen on neurological outcome following Brain Ischemia During post-cardiac arrest care (HYBRID II trial): study protocol for a randomized controlled trial	
Arola et al. (56) Fries et al. (55)	Argon and Xenon	Human/inhalation Pig/inhalation	Anti-Apoptotic	Effect of Xenon and Therapeutic Hypothermia, on the Brain and on Neurological Outcome Following Brain Ischemia in Cardiac Arrest Patients (Xe-hypotheca) Significant improvements in functional recovery and ameliorated myocardial dysfunction	

*IV, intravenous; IP, intraperitoneal; SQ, subcutaneous*.

The use of combined therapy of adenosine triphosphate-magnesium chloride (ATP-MgCl_2_), norepinephrine, and vanadate improved protein synthesis and conferred neuroprotection after CA and resuscitation in rats as compared to individual drug treatment (108). Norepinephrine counteracts the vasodilatory effects of ATP and maintains blood pressure resulting in a selective vasodilatory effect in the brain as a result of this combination therapy. In ventricular fibrillation cardiac arrest (VFCA) rats, a combination therapy of niacin and selenium reduced ROS generation by enhancing glutathione (GSH) reductase activity and improved the GSH/GSSG ratio, attenuated brain injury, and improved the 7-day neurological outcomes by suppression of mechanisms that would normally increase caspase-mediated cell death (109). Another major limitation in these combination therapies is the use of very mild CA injury in the animals that does not reflect the injury of many human CA patients. A different combination of sevoflurane, Poloxamer-188, and TTM improved cardiac and neurologic functions after 17 min VF in a swine model as compared with controls that only received epinephrine and TTM (110). It is suggested that sevoflurane promotes endothelial protection by reducing leukocyte activation, affecting vascular tone (111), preventing apoptosis, and reducing cytokine production post-CA (112). Poloxamer-188 fills ischemia induced pores in the plasma membrane (113), prevents unregulated exchange of ions between cellular compartments, prevents cellular injury and apoptosis (114), preserves the BBB (115), and protects neurons against IRI (116). A major limitation with this combination is the lack of mechanistic insight of the pharmacologic compounds which is further complicated by TTM and the lack of dose optimization.

The combination therapy of epinephrine and vasopressin has shown potential beneficial effects in improving ROSC, and/or neurological and cerebral histopathological outcomes in animal models; however, this combination has only mixed results in the clinical setting (117–120). As potent vasoactive compounds, the potential adverse hemodynamic effects especially after a severe injury like CA are important considerations when combining these drugs, which may be one reason for the lack of translatability. A randomized control trial in humans with the combination of epinephrine, vasopressin, and methylprednisolone post-CA compared to epinephrine and saline demonstrated improved survival to hospital discharge with favorable neurological status (121–123). In fact, the singular administration of steroids during the post-arrest period has been associated with diminished brain enzyme changes, decreased requirement for vasopressor, and improved EEG and cardiovascular recovery in rodent CA (105) and improved survival to discharge in human patients (104, 124). The multisystemic effects of steroids present difficulty in isolating individual therapeutic actions, which can include hemodynamic, metabolic, and inflammatory modulation. The combination of vasopressin, epinephrine, and nitroglycerin has been shown to improve vital organ blood flow in pigs (125). Combination of epinephrine and naloxone significantly improved the survival and brain function post-CA in rats (126, 127). These studies emphasize that pharmaceutically managing hemodynamics during the resuscitation and post-resuscitation phases is critical for survival and protecting the brain.

Along with hemodynamic stabilization after arrest to maintain brain perfusion, managing the metabolic dysfunction is another critical requirement for post-CA cocktail therapy. The current, most effective method used is TTM, that is hypothesized to decrease the global metabolic demand, which is not without adverse effects (128). A new approach to achieve dampened metabolic stress post-arrest is to pharmacologically induce hypothermia (129). A combination of ethanol, vasopressin, lidocaine, known as HBN-1, after rodent CA was able to pharmacologically induce hypothermia by increasing heat loss without producing shivering and improve survival and neurological outcomes (95, 130). Similarly, HBN-1 with external hypothermia was shown to significantly decrease serum and cerebrospinal fluid levels of neuron specific enolase (NSE), a biomarker associated with poor neurologic outcomes (131). As is evident from previous preclinical and clinical post-CA trials, combination drugs have many benefits by simultaneously targeting different altered pathways; however, due to the severity of injury experienced by the whole body, a multi-drug cocktail should be similarly extensive in its therapeutic agents to appropriately manage the disease process.

## Discussion

The global ischemia after CA results in a myriad of systemic insults, such as mitochondrial dysfunction (17), increased reactive oxygen species (ROS) generation (17, 18), metabolic alterations including lipid and protein dysfunction (19–21), along with other pathological sequelae. Although various single drug and some combination therapies have shown neuroprotection, to date, the overall outcomes have not been substantially improved. One potential reason is the lack of translatability of many of these agents to human patients; it is known that animal models are helpful in modeling disease, however, there are many challenges and limitations, especially when attempting to model a condition with a complex pathophysiology (19). Most preclinical studies use milder injury CA models that are unable to effectively represent the magnitude of human disease (132). Another explanation is that using singular drugs may effectively target the intended pathway, but are unable to alleviate the plethora of altered mechanisms by which brain damage occurs post-CA. A new shift in CA treatment can entail the incorporation of a multi-drug cocktail comprising a variety of drugs that can individually and synergistically confer neuroprotection based on their effects on the aforementioned diverse pathophysiological sequelae of CA. Furthermore, with the protective effects of TTM and E-CPR in eligible CA patients (9), it is conceivable that a cocktail therapy may provide additive benefits when combined with conventional interventions (14). Figure 1B highlights the various dysregulated pathways in the brain post-CA, and the respective interventions required for appropriate management that can be included in a cocktail therapy.

The important components for developing a cocktail therapy includes the types of pharmaceutical agents, formulations, dosages, modes of delivery and pharmacodynamics, as well as the potential translatability to human patients. One study of prolonged VFCA in pigs found that direct administration of the combination of epinephrine, vasopressin, amiodarone, sodium bicarbonate, and metoprolol worsened short-term outcomes as compared to serial administration (133). The treatment of CA involves a complex pathology at the arrest, ROSC, and the post-ROSC phases. Therefore, ideally, a cocktail therapy should be phase-based as well as incorporating the general parameters, such as types of drugs, dosages, and methods of administration. The phase-based approach of cocktail development implies that the cocktail comprises of drugs that during ROSC and post-ROSC phases can (1) stabilize hemodynamics, (2) maintain mitochondrial membrane and function integrity, (3) decrease ROS generation, (4) provide metabolic supplementation, (5) reduce neuronal excitotoxicity, and (6) modulate neuroinflammation and protect blood-brain barrier integrity.

The concept of cocktail therapy has been applied in both preclinical and clinical settings for stroke to target various altered ischemic cascades with and without TTM (134–139). Although the focal IRI in stroke contrasts the global brain ischemia and damage observed in CA, the general neuronal protection observed in stroke combination therapies may be directly utilized, supplemented with other agents, and repurposed in a cocktail treatment of brain injury post-CA. Targeting multiple pathways allows for maximal neuroprotection, as targeting solitary pathways does not address the other dysregulated pathways that cause organ damage. Ultimately, while a combination of drugs is needed to mitigate the damage post-CA, the drugs chosen should be mechanistically multi-functional in order to use the fewest number of drugs possible to create the most efficacious multi-drug cocktail.

## Conclusion

This review has summarized the current landscape of CA interventions using single drug therapy or a few drug combination therapies to target one or a few pathways to potentiate neuroprotection in both the preclinical and clinical environments. Furthermore, the implicated pathways of CA pathophysiology are the targets of various interventions and provide a foundation for the development of an extensive multi-drug cocktail; the use of a cocktail, involving several pharmaceutical agents, can simultaneously target the multitude of altered pathways and may synergistically confer neuroprotection. This cocktail may be further combined with more advanced resuscitation procedures, such as TTM and E-CPR. A cocktail therapy that combines various studied agents that can stabilize the hemodynamic, mitochondrial, metabolic, oxidative, and inflammatory processes may aid in reducing brain injury after cardiac arrest and improve survival with favorable neurologic outcomes.

## Author Contributions

RC, MS, and LB contributed to the conception of the review and edited and revised the manuscript critically for important intellectual content. MS, SS, and RC contributed to the writing of the paper and interpreting relevant literature. DR, DJ, SM, KH, EM, and JK edited and revised the manuscript critically for important intellectual content. All authors contributed to the article and approved the submitted version.

## Conflict of Interest

LB has a grant/research support from Philips Healthcare, the NIH, Nihon Kohden Corp., Zoll Medical Corp, PCORI, BrainCool, and United Therapeutics and owns several issued and pending patents involving the use of medical slurries as human coolant, devices to create slurries, reperfusion cocktails, and measurement of respiratory quotient. The remaining authors declare that the research was conducted in the absence of any commercial or financial relationships that could be construed as a potential conflict of interest.

## Glossary

ACA: asphyxial cardiac arrest

ACLS: advanced cardiovascular life support

AMPK: 5′-adenosine monophosphate-activated protein kinase

ATP-MgCl_2_: adenosine triphosphate-magnesium chloride

CA: cardiac arrest

CL: cardiolipin

CPB: cardiopulmonary bypass

CPR: cardiopulmonary resuscitation

DETC-MeSO: S-Methyl-N, N-diethylthiocarbamate sulfoxide

DRP1: dynamin-related protein 1

E-CPR: extracorporeal cardiopulmonary resuscitation

eIF2α: eukaryotic Initiation Factor 2 alpha

ER: endoplasmic reticulum

GBC: glibenclamide

HIF-1α: hypoxia-inducible factor 1-alpha

IHCA: in-hospital cardiac arrest

IRI: Ischemia- Reperfusion Injury

IL1-α: interleukin-1 alpha

IL-1β: interleukin-1 beta

IL-18: interleukin-18

LPC: lysophosphatidylcholine

LPE: lysophosphatidylethanolamine

LPI: lysophosphatidylinositol

MCAO: middle cerebral artery occlusion

Mdivi-1: mitochondrial division inhibitor 1

MLCL: monolysocardiolipin

mPTP: mitochondrial permeability transition pore

NDS: neurodeficit score

NF-κB: nuclear factor-kappa B

NLRP3: nucleotide-binding oligomerization domain, leucine rich, repeat and, pyrin domain-containing 3

NMDA: N-Methyl- d-aspartate

NSE: neuron specific enolase

OHCA: out-of-hospital cardiac arrest

PDH: pyruvate dehydrogenase

ROS: reactive oxygen species

ROSC: return of spontaneous circulation

S1QEL: suppressor of site IQ electron leak

SS-31: Szeto-Schiller peptide-31

TCA: tricarboxylic acid

TH: therapeutic hypothermia

TNF-α: tumor necrosis factor alpha

TTM: targeted temperature management

UPR: unfolded protein response

VFCA: ventricular fibrillation cardiac arrest
